# Absence seizures in lesion-related epilepsy

**DOI:** 10.1186/s42494-023-00133-4

**Published:** 2023-09-13

**Authors:** Xiaoqin Sun, Miao Wang, Zeng He, Lihong Liu, Xianjun Shi, Chunqing Zhang, Ning An, Meihua Yang, Zhifeng Wu, Ruodan Wang, Li Wang, Zhongke Wang, Hui Yang, Xiaolin Yang, Shiyong Liu

**Affiliations:** 1 Department of Neurosurgery, Second Affiliated Hospital, Army Medical University, 183 Xinqiao Main Street, Shapingba District, Chongqing, 400037 China; 2 Department of Pediatrics, Second Affiliated Hospital, Army Medical University, 183 Xinqiao Main Street, Shapingba District, Chongqing, 400037 China; 3 Department of Neurology, Second Affiliated Hospital, Army Medical University, 183 Xinqiao Main Street, Shapingba District, Chongqing, 400037 China; 4 Department of Neurosurgery, Armed Police Hospital, Chongqing, 400037 China; 5 Chongqing Institute for Brain and Intelligence, Guangyang Bay Laboratory, Chongqing, 400037 China

**Keywords:** Absence seizure, Lesion-related epilepsy, Focal epilepsy, Lennox-Gastaut syndrome

## Abstract

**Background:**

In the new International League Against Epilepsy (ILAE) classification of seizure types, generalized seizures such as absence seizures (ASs) may originate from a focal point and rapidly spread to the bilaterally distributed brain network. Increasing evidence from animal and clinical studies has indicated that focal changes may occur prior to ASs; however, the relationship of ASs with epileptogenic lesions remains unclear.

**Methods:**

We retrospectively collected and analyzed the clinical, imaging, and electrophysiological data of 16 patients who had ASs and structural lesions with seizure-free outcomes after lesion resection.

**Results:**

In semiology analysis, nine patients displayed focal onset; only two patients showed simple ASs, and seizure types other than ASs were observed in the remaining patients. On ictal electroencephalography (EEG), four patients showed bilateral synchronous symmetric 3 Hz generalized spike-wave discharges (GSWDs), and the remaining patients showed bilateral 1.5–2.5 Hz GSWDs. Moreover, most patients (13/16, 81.3%) exhibited focal features in addition to ASs, while interictal EEG was the same in 12 patients. Furthermore, on stereoelectroencephalogram (SEEG), 2/5 patients showed focal discharges before bilateral burst GSWDs. Additionally, all patients had structural lesions on imaging. In four typical AS patients, the lesions were located in deep brain regions. Notably, in 9 patients (9/16, 56%), the lesions were located in the posterior cortex. All patients underwent lesion resection and had seizure-free outcomes during follow-up, and intelligence quotient (IQ) also improved by 10.71 ± 3.90 one year after surgery.

**Conclusions:**

Patients with lesion-related epilepsy may present with ASs that have a focal onset and are associated with good surgical outcomes.

## Background

Absence seizures (ASs) are characterized by the transient disruption of consciousness related to sudden cessation of activity and a highly recognizable electrographic pattern of 3 Hz generalized (bilateral, symmetric, and synchronous) spike-wave discharges (GSWDs), which are commonly present in childhood absence epilepsy (CAE), juvenile absence epilepsy (JAE), epilepsy with myoclonic absences, eyelid myoclonia with absences and other epilepsy or epilepsy syndromes [1–3]. Compared with typical absence seizures, atypical absence seizures start slowly, triggering a mild disturbance of consciousness, and may be accompanied by tonic, atonic, myoclonic and 1.5–2.5 Hz GSWDs, which are also well-known patterns of Lennox-Gastaut syndrome (LGS) [4, 5]. According to the International League Against Epilepsy (ILAE) classification from 2017, ASs are classified as a form of generalized epilepsy and are recognized as difficult to treat surgically [6]. However, it has been reported that patients with ASs have focal seizures in addition to absence seizures [4, 7, 8], and LGS patients with focal lesions and atypical ASs have favorable surgical outcomes [9, 10], suggesting that ASs occur in patients with lesion-related epilepsy, while their specific electroclinical characteristics and relationship are still unknown.

Although there are many hypotheses regarding the mechanisms underlying absence seizure generation, the abnormal oscillation rhythm of the thalamocortical circuit is currently thought to be critical in its pathogenesis [1, 11, 12]. Epileptic discharges at any point in the thalamocortical pathway may activate the whole circuit and cause rhythmic oscillations, which further suggests that the cortical focus is involved in the generation of ASs [13]. Furthermore, increasing evidence demonstrates that focal lesions are observed in patients with ASs, and focal electrophysiological changes prior to ASs have been detected [1, 5, 11]. These findings indicate that absence seizures may be related to focal brain lesions, although focal changes are significantly different from typical focal seizures. For this reason, we collected data from a cohort of patients with lesion-related epilepsy who had ASs (electroencephalography showed 3 Hz GSWDs or 1.5–2.5 Hz GSWDs) and a good surgical outcome after lesion resection at the Epilepsy Centre of Xinqiao Hospital within the past 10 years (July 2011 to June 2021). The results showed a close relationship between lesions and absence seizures.

## Methods

### Subjects

**Fig. 1:**
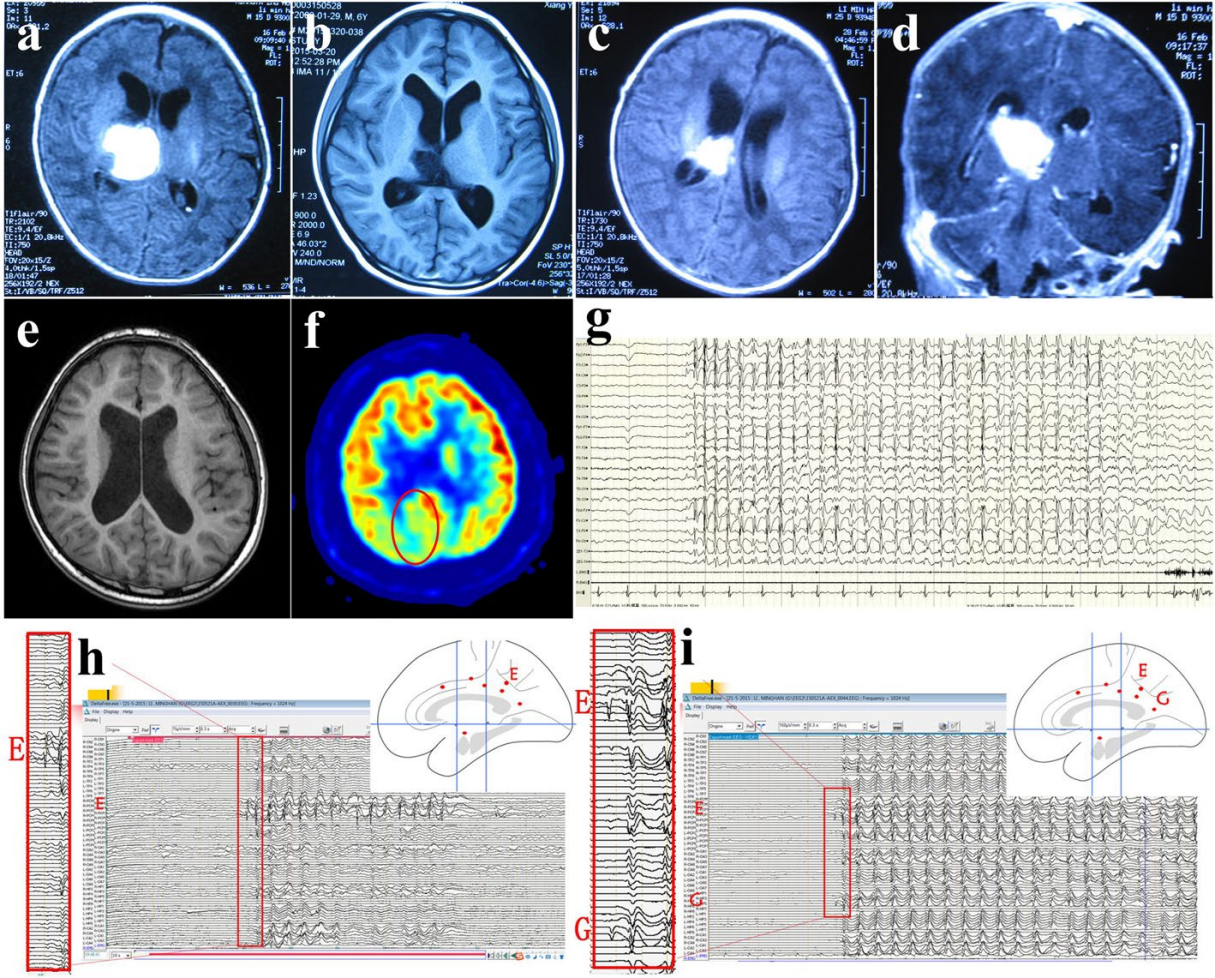
Clinical characteristics of a typical absence seizure patient (No. 14 in Table 1). **a** Hemorrhagic foci in the right thalamus due to vitamin K1 deficiency at 7 days after birth. **b** Malacia in the right thalamus; **c**-**d** hemorrhage involved the posterior cingulate gyri. **e-f** No obvious abnormality was observed in the posterior cingulate gyrus before surgery (**e**), but PET showed a hypometabolism focus in the medial parietal lobe and posterior cingulate gyri (**f**). **g** Scalp EEG showed bilateral synchronous 3 Hz GSWDs during absence seizures. **h**-**i** SEEG showed interictal discharges obviously originating from the right posterior cingulate cortex (electrodes E1-4) (**h**) and ictal focal changes (electrodes E1-4) before GSWDs during absence seizures (**i**)

**Fig. 2:**
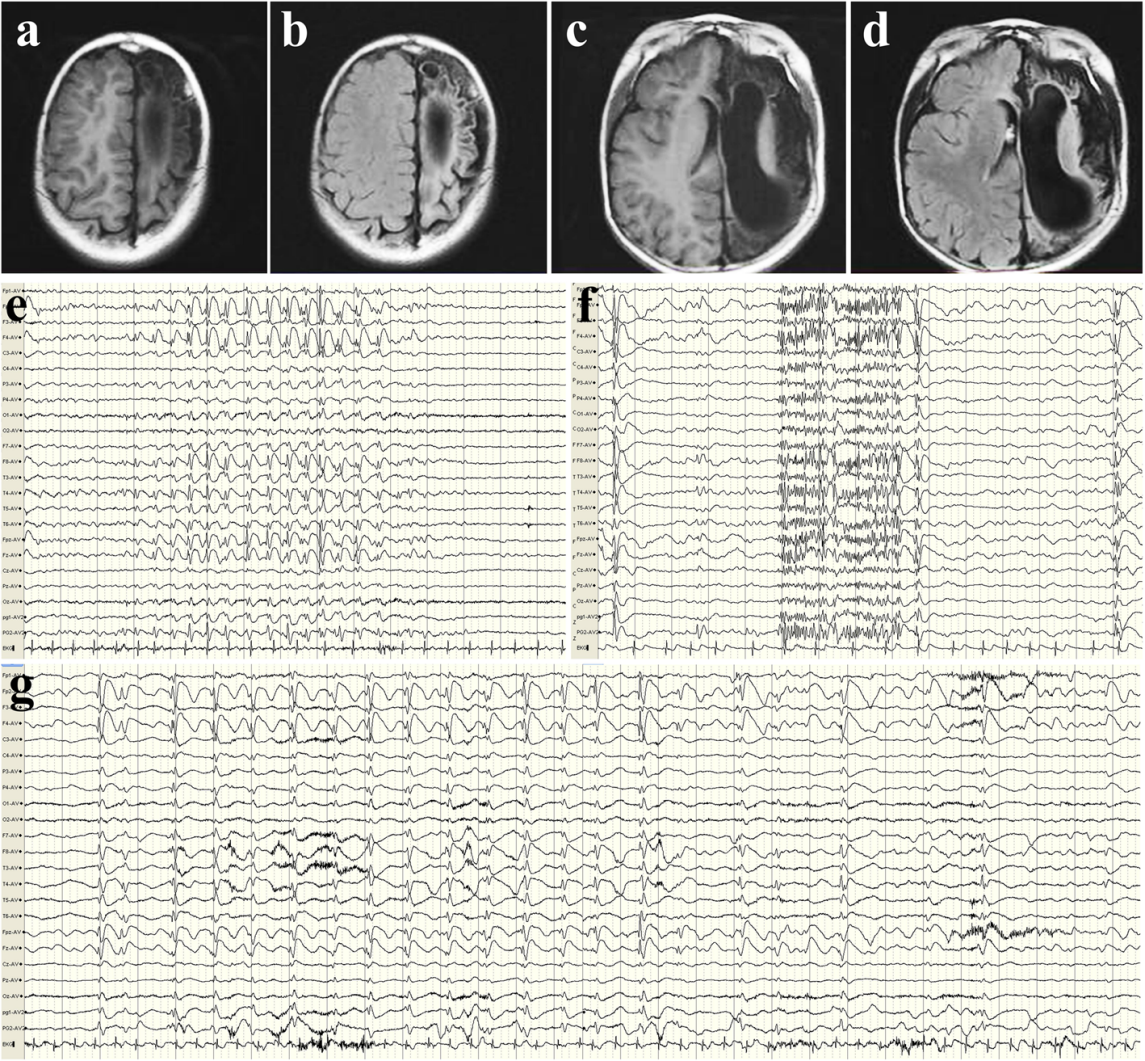
A case of atypical absence seizures in LGS (No. 1 in Table 1). **a**-**d** Intracranial hemorrhage caused by trauma at 12 days after birth, with subsequent encephalomalacia. **e** Interictal asymmetric slow-spike and wave discharges (SWDs) are obvious on the right. **f** EEG showed a paroxysmal fast activity (PFA) pattern during sleep. **g** Scalp EEG showed 1.5–2.5 Hz asymmetric SWDs during atypical absence seizures

A retrospective analysis was performed on patients at the Epilepsy Centre of Xinqiao Hospital who underwent epileptogenic lesion resection from July 2011 to June 2021 and were confirmed by scalp video electroencephalography (VEEG) to have ASs. ASs manifest as (1) typical absence seizures, with a sudden cessation of activity and transient disruption of consciousness (4 - 30 s) [14], accompanied by 3 Hz generalized (bilateral, symmetric, and synchronous) spike-wave discharges (GSWDs) (Fig. 1) or (2) atypical absence seizures, which may start slowly and trigger a mild disturbance of consciousness and may be accompanied by tonic, atonic, myoclonic and 1.5–2.5 Hz GSWDs, and their rhythms can be irregular, heterogeneous, and perhaps mixed with fast rhythms (Fig. 2) [14], and (3) there is no focal semiology or scalp electrophysiological origin when the absence seizure occurs. The inclusion criteria were as follows: (1) absence seizure confirmed by presurgical evaluation; (2) intractable epilepsy with MRI structural lesions treated by surgical resection; and (3) follow-up for at least 1 year and seizure free after resection (ILAE class I outcome), the purpose was to reconfirm the significant association between focal epileptic focus and ASs and exclude possible confounding factors, such as uncertain lesions and inadequate extent of resection. The exclusion criteria were as follows: (1) blank staring without GSWDs; (2) insufficient follow-up data or follow-up of less than one year; and (3) seizures that persisted after the operation (early seizures within 1 month after operation were not included in the exclusion criteria).

**Table 1:** The detailed clinical information of patients

No	Gender	Age at surgery (y)	Age at seizure onset (y)	Seizure types	Focal semiology	Seizure frequency	Etiology	IQ	Preoperative anti-seizure medications	Scalp EEG	SEEG	MRI	PET	Surgery	Postoperative EEG	outcome (ILAE class)	Histopathologic findings
1	Male	5	3	LGS: spasms/tonic/ focal /atypical absence	Right upper limb tonic	every day	Structural (Malacia)	42	OXC, VPA, LEV, TPM	Interictal: asymmetry SWD, paroxysmal fast activities, generalized discharge; Ictal: focal (right hemisphere) & generalized	-	Encephalomalacia of the left hemisphere	Hypometabolism of the left hemisphere	Left hemispherectomy	ED disappeared	1	Nonspecific gliosis
2	Female	13	3	LGS: spasms/tonic/ focal /atypical absence/focal to bilateral tonic-clonic seizures	Left upper limb tonic and, turning of head and eyes to the left	per week	Structural (Ischemic encephalatrophy)	56	LTG, LEV, OXC	Interictal: asymmetry SWD, paroxysmal fast activities, posterior discharge; Ictal: focal (right hemisphere) & generalized	-	Right parietal-occipital encephalomalacia	Hypometabolism at right temporal lobe, parietal lobe, occipital lobe	Right occipital and posterior temporal resection	ED remarkably improved	1	Nonspecific gliosis
3	Male	6	0.2	LGS: focal spasms/tonic/atypical absence	Head nodding to the left	every day	Structural (Malacia after intracerebral hemorrhage)	25	VPA, CZP, TPM	Interictal: asymmetry SWD, paroxysmal fast activities, anterior cortex discharge; Ictal: focal (right frontal) & generalized	-	Encephalomalacia of the right hemisphere	-	Right hemispherectomy	ED disappeared	1	Nonspecific gliosis
4	Male	3	0.2	LGS: spasms/tonic/atypical absence/focal to bilateral tonic-clonic seizures	Turning of head and eyes to the left	every day	Structural (Malacia)	-	OXC, VPA, LTG	Interictal: asymmetry SWD, paroxysmal fast activities, generalized discharge; Ictal: focal (left hemisphere) & generalized	-	Encephalomalacia of the right hemisphere	-	Right subtotal hemispherectomy	ED remarkably improved	1	Nonspecific gliosis
5	Male	12	6	LGS: spasms/tonic/atypical absence	-	every day	Structural (FCD)	42	VPA, CZP, TPM	Interictal: GSWD, paroxysmal fast activities, right frontal focal discharge; Ictal: focal (right frontal) & generalized	Tonic and spasm seizures: originate from medial right frontal lobe	Right frontal FCD	Right frontal hypometabolism	Right frontal partial resection	ED disappeared	1	FCDIIA
6	Male	7	4	LGS: spasms/ focal tonic/atypical absence	Left upper limb tonic	every day	Structural (Ischemic malacia)	37	OXC, VPA, LEV	Interictal: asymmetry SWD, paroxysmal fast activities, right parietal focal discharge; Ictal: focal ( right parietal) & generalized	-	Encephalomalacia of the left occipital lobe	Hypometabolism at right temporal lobe, parietal lobe and occipital lobe	Right parietal-occipital resection	ED remarkably improved	1	Nonspecific gliosis
7	Male	6	4	LGS: tonic/atypical absence/focal	Turning of tonic head to the right	every day	genetic &structural (Tuberous sclerosis complex)	71	LEV, VPA, OXC	Interictal: GSWD, paroxysmal fast activities, anterior cortex discharge. Ictal: focal (left hemisphere) & generalized	-	Multiple cortical tubers and subependymal nodules	Multiple hypometabolism in bilateral hemisphere	Resection of left frontal tuber and the surrounding tissue	ED remarkably improved	1	FCDII
8	Female	23	2	LGS: spasms/tonic/GTCS/atypical absence	-	Per week	Structural (Ischemic encephalatrophy)	52	LTG, LEV, VPA	Interictal: asymmetry SWD, paroxysmal fast activities; Ictal: focal (left temporal-parietal) & generalized	-	Left temporal-parietal atrophy	Hypometabolism at left temporal lobe, parietal lobe	Left temporal-parietal resection	ED improved, but not remarkably	1	Nonspecific gliosis
9	Male	15	4	LGS: spasms/atypical absence	-	every day	Structural (FCDII)	56	VPA, CBZ, TPM	Interictal: GSWD, paroxysmal fast activities, generalized discharge; Ictal: focal (left frontal) & generalized	spasm seizure: originate from left lateral frontal cortex	Left frontal FCD	Left frontal hypometabolism	Left frontal resection	ED remarkably improved	1	FCDIIA
10	Female	7	5	Non-LGS: focal to bilateral tonic-clonic seizures/atypical absence;	Left upper limb tonic	every day	Structural (Malacia)	25	LCM, VPA, LTG	Interictal: right parietal-occipital discharge; Ictal: focal (right parietal-occipital) & generalized	Focal to bilateral seizure: originate from right medial occipital cortex	Right temporal-parietal-occipital encephalomalacia	Hypometabolism at right temporal lobe, parietal lobe, occipital lobe	Right temporal-parietal-occipital resection	ED remarkably improved	1	Nonspecific gliosis
11	Male	5	2	Non-LGS: focal to bilateral tonic-clonic seizures/atypical absence	Right upper limb tonic	every day	structural (FCD)	68	OXC, TPM, VPA	Interictal: focal left central-parietal discharge; Ictal: focal (left central-parietal) & generalized	-	Left parietal FCD	Hypometabolism at left temporal lobe, parietal lobe	Left parietal resection	ED disappeared	1	FCDIIB
12	Female	10	8	Non-LGS: focal /typical absence	Turning of head to the left	Per week	Structural (FCD)	76	LTG, LEV, VPA	Interictal: right posterior discharge; Ictal: focal (right posterior) & generalized	-	Right medial parietal FCD	Right medial parietal hypometabolism	Right medial parietal resection	ED disappeared	1	FCDIIA
13	Female	14	10	Non-LGS: GTCS, typical absence	-	every day	Structural (FCD)	78	TPM, VPA, CBZ	Interictal: generalized left medial parietal discharge; Ictal: generalized	GTCS: originate from left posterior cingulated cortex; absence seizures: originate from left posterior cingulated cortex, and rapidly engaging, GSWD	Left posterior cingulated FCD	Left parietal Hypometabolism	Left medial parietal (including posterior cingulated cortex)resection	ED disappeared	1	FCDIIA
14	Male	6	3	Non-LGS: typical absence	-	every day	Structural (Right thalamic malacia)	46	LTG, LEV, CZP	Interictal: generalized right medial parietal discharge; Ictal: generalized	absence seizures: originate from right posterior cingulated cortex, and rapidly engaging, GSWD	Right thalamic malacia	Right medial parietal hypometabolism	Right medial parietal ( including cingulated cortex ) resection	ED remarkably improved	1	nonspecific gliosis
15	Female	7	3	Non-LGS: myoclonic /spasms/tonic/atypical absence	-	Per week	genetic structural (FCD)	38	VPA, LTG, TPM, PB	Interictal: Left parietal, occipital and posterior temporal discharge; Ictal: focal (left temporal, parietal) & generalized	-	Left posterior FCD	Hypometabolism at left temporal lobe, parietal lobe, occipital lobe	Left temporal, parietal and occipital resection	ED disappeared	1	FCDIIA
16	Male	7	5	Non-LGS: typical absence	-	every day	Structural (FCD)	82	VPA, TPM	Interictal: bilateral central-parietal discharge; Ictal: generalized	-	Right insular lobe FCD and adjacent arachnoid cyst	Hypometabolism at right temporal lobe	resection of the right temporal lobe	ED remarkably improved	1	FCDIA

*Abbreviations*: *ED *Epileptic discharges, *IQ *Intelligence Quotient, *LGS *Lennox–Gastaut syndrome, *FCD *Focal cortical dysplasia, *GSWD *Generalized spike-wave discharges, *OXC *Oxcarbazepine, *VPA *Valproic acid, *LEV *Levetiracetam, *TPM *Topiramate, *LTG *Lamotrigine, *PB *Phenobarbital, *CBZ *Carbamazepine, *LCM *Lacosamide, *CZP *Clonazepam, *EEG *Electroencephalography, *SEEG *Stereoelectroencephalography, *MRI *Magnetic resonance imaging, *PET *Positron emission tomography, *ILAE *International League Against Epilepsy

### Presurgical evaluation

Noninvasive presurgical evaluation included a medical history review, neurological examination, thin-section MRI, 18 F-FDG-PET, long-term VEEG recording, and neuropsychological tests. MRI scans included 1.5 T/3.0 T precontrast axial T1-weighted imaging (T1WI) and T2-weighted imaging (T2WI), diffusion-weighted imaging (DWI), sagittal T1WI, and 3D-T2 FLAIR. Continuous VEEG monitoring was performed in all patients prior to surgery, and VEEG data were recorded using a digital electroencephalogram (EEG) machine (Nihon Kohden or Nicolet). Scalp EEG electrodes were installed according to the 10–20 electrode system of the International Federation of Electroencephalography. Sphenoid electrodes and activation were also utilized in some patients. At least three habitual seizures were captured, and two physicians specializing in EEG independently analyzed the interictal and ictal events documented by VEEG. In addition, subjects completed psychological assessments such as the Wechsler Intelligence Scale (Gesell Developmental Schedules for children under 4 years old) and the Quality of Life in Epilepsy-31 (QOLIE-31; QOLIE-76 for children).

Subjects who could not be identified by noninvasive preoperative evaluation were implanted with a stereoelectroencephalogram (SEEG) electrode for seizure localization. Electrodes (HKHS Healthcare Co., Ltd., Beijing, China; 16 contacts) were implanted under robotic guidance. SEEG data were recorded using a 128- or 256-channel system from Japan, filtered between 0.1 and 600 Hz and sampled at 2000 Hz. The epileptogenic focus was determined by the mutual coincidence of detailed history, video-EEG recording, MRI, and PET. When these findings were contradictory, they were combined with the results of SEEG.

### Analysis of VEEG and imaging

Two experienced epileptologists visually reviewed the EEG data. Seizure types were diagnosed by a comprehensive evaluation of video EEG, technician bedside observation, patient or family description, and clinical history. The distribution of epileptiform discharges on interictal and ictal EEG was recorded to analyze their relationship with the epileptogenic focus. For ASs, the onset, evolution, and duration of symptoms during the ictal period and their relationship with EEG were specifically studied. The MRI and PET results were based on the diagnosis determined by two radiologists and/or a senior epilepsy surgeon.

### Surgery and follow-up

Different types of surgical resections or dissections, including lesion resection, lobe resection and hemispherectomy or dissection, were performed based on presurgical evaluation and electrocorticography (ECoG) monitoring during the operation. After surgery, anti-seizure medication (ASM) was maintained for at least 2–3 years, and follow-up was conducted every 3–6 months, including neuropsychological tests performed 1–2 years after surgery.

### Statistical analysis

Counting data are expressed as the mean ± SD. Data analysis was performed using Student’s *t* test, Mann‒Whitney U test or chi-square test. Statistical significance was set at *P* < 0.05. All statistical analyses were performed using SPSS 22.0.

## Results

### General information

According to the inclusion and exclusion criteria, 16 patients (10 males and 6 females) representing 0.76% (16/2113) of the epilepsy patients treated from July 2011 to June 2021 were enrolled in the study. The age of seizure onset was 3.90 ± 1.85 years (0.2–10 years), the duration was 5.16 ± 3.53 years (1.5–21 years), and the age at surgery was 9.13 ± 4.03 years (3–23 years). The average presurgical intelligence quotient (IQ) was 52.93 ± 12.25, and the average number of presurgical medication type was 3.06. Detailed patient information is presented in Table 1.

### Semiology of absence seizures

All patients presented with ASs, of which 4 were diagnosed as typical and the remaining 12 were classified as atypical. Accompanying semiologies included mild staring (10 patients), nodding before absence seizures (3 patients), oropharyngeal automatisms (2 patients), blinking (2 patients), mild shaking of limbs (2 patients), and mild tonic (1 patient). Seizures occurred every day in 12 patients (12/16, 75%), while the remaining patients experienced seizures per week. Seizure durations also varied, ranging from 4 to 10 s in 7 patients, 10–20 s in 6 patients and 20–60 s in 3 patients (Table 2).

**Table 2:** The characteristic of absence seizure in the study

No.	Main symptoms of seizure	Accompanied symptoms of seizures	seizure duration	Seizure frequency	EEG background	Ictal EEG	SEEG	Manifestation of absence seizure	Genetic testing
1	Staring, sudden cessation of activity, transient disruption of consciousness.	Nodded before seizure	10s	1–2/day	Background abnormalities(left lazy activity, generalized θ activity)	1.5-2.5 Hz GSWD, low amplitude on the left	-	Spasms-atypical absence seizure	-
2	Staring, sudden cessation of activity, transient disruption of consciousness	Nodded before seizure, and swallowing	45-60s	2–3/week	Mild background abnormalities	2 Hz GSWD	-	Spasms atypical absence seizure	Negative
3	Sudden cessation of activity, transient disruption of consciousness	-	9-10s	1–2/day	Background abnormalities(generalized θ/δ activity)	1.5-2.5 Hz GSWD	-	Atypical absence seizure	-
4	Staring, sudden cessation of activity, transient disruption of consciousness	Mild shaking of limbs or swallowing	10s	1/day	Background abnormalities(diffuse θ/δ activity, right lazy activity)	1.5-2.5 Hz GSWD, low amplitude on the right	-	Atypical absence seizure	-
5	Staring, sudden cessation of activity, transient disruption of consciousness	-	10-15s	1–2/day	normal background	1.5-2.5 Hz GSWD	Absence seizure was not monitored	Atypical absence seizure	Negative
6	Sudden cessation of activity, transient disruption of consciousness	-	10-14s	1–2/day	Background abnormalities(diffused θ/δ activity)diffused theta/delta activity	1.5-2.5 Hz GSWD, predominance of left frontotemporal	-	Atypical absence seizure	-
7	Staring, sudden cessation of activity, transient disruption of consciousness	-	10s	1–2/day	Background abnormalities(diffused θ/δ activity)	1.5-2.5 Hz GSWD	-	Atypical absence seizure	*TSC2*
8	Staring, sudden cessation of activity, transient disruption of consciousness.	-	16-17s	2–3/week	Mild background abnormalities	2.5 Hz GSWD	-	Atypical absence seizure	-
9	Staring, sudden cessation of activity, transient disruption of consciousness.	-	5-6s	1/day	Mild background abnormalities	1.5-2.5 Hz GSWD	Atypical absence seizures: generalized SWD	Atypical absence seizure	Negative
10	Staring, sudden cessation of activity, transient disruption of consciousness.	Nodded before seizure	20-25s	1/day	Background abnormalities(diffused θ/δ activity)	1.5-2 Hz GSWD(obvious on posterior cortex)	Spasms-atypical absence seizure: start from right lateral occipital cortex→GSWD	Spasms-atypical absence seizure	Negative
11	Staring, sudden cessation of activity, transient disruption of consciousness.	Blinking	5-9s	2–3/day	Background abnormalities(diffused θ/δ activity)	2-2.5HzGSWD, left parietal-occipital present first	-	Atypical absence seizure	-
12	Sudden cessation of activity, transient disruption of consciousness.	-	7-9s	3–5/week	Normal background	3 Hz GSWD	-	Typical absence seizure	Negative
13	Sudden cessation of activity, transient disruption of consciousness.	Mild shaking of limbs	6-21s	5–6/day	Normal background	3 Hz GSWD	Typical absence seizure: start from left posterior cingulated cortex, rapidly engaging GSWD	Typical absence seizure	Negative
14	Sudden cessation of activity, transient disruption of consciousness.	-	11-16s	3–4/day	Normal background	3Hz GSWD	Typical absence seizure: start from right posterior cingulated cortex, rapidly engaging GSWD	Typical absence seizure	Negative
15	Staring, sudden cessation of activity, transient disruption of consciousness.	Mild tonic	40-50s	1/week	Background abnormalities(diffused θ rhythm)	1.5-2.5 Hz GSWD	-	Atypical absence seizure	*NPRL2*
16	Sudden cessation of activity, transient disruption of consciousness.	Blinking	9-15s	5–6/day	Normal background	3 Hz GSWD	-	Typical absence seizure	Negative

*GSWD *Generalized spike-wave discharges, *EEG *Electroencephalography, *SEEG *Stereoelectroencephalography

According to a previous study, we categorized abnormal EEG background as mild, moderate or severe [15]. The EEG background was normal in 5 patients, mildly abnormal in 3 patients, and severely abnormal in 8 patients (generalized slow rhythm in most patients and unilateral lazy waves in 2 patients). On ictal EEG, four subjects exhibited bilateral synchronous symmetric 3 Hz GSWDs, and the remaining subjects exhibited bilateral generalized 1.5–2.5 Hz GSWDs (bilateral asymmetry in 5 subjects). SEEG recordings were conducted in five subjects, four of whom demonstrated ASs, while two patients with simple AS exhibited focal discharges preceding bilateral burst GSWDs (Fig. 1). Imprinted gene detection was performed in ten subjects, and two patients tested positive for mutations in the *TSC2* and *NPRL2* genes. Details are provided in Table 2.

### Clinical manifestations of other accompanying types of seizures

In addition, 87.5% of patients (14/16) exhibited seizure types other than ASs, including focal seizures or focal-to-bilateral tonic‒clonic seizures in 9 patients (including one case of focal tonic seizures and one case of focal epileptic spasms). Of the 14 patients, 12 showed focal or unilateral discharge on interictal scalp EEG, and 13 showed focal or unilateral onset on ictal scalp EEG. In five patients who underwent SEEG recording, three patients showed focal seizures and absence seizures, and the other two patients presented with simple ASs.

### Neuroimaging

All subjects showed structural lesions on MRI. Eight patients had encephalomalacia or encephalatrophy (five cases were local and three cases were hemispheric), while the other eight patients showed focal cortical dysplasia (FCD). One of these cases was diagnosed as tuberous sclerosis complex (TSC) due to the combination of cutaneous findings and a *TSC2* gene mutation, with multiple cortical tubers and subependymal nodules present. Another case was accompanied by an arachnoid cyst. Four patients with typical ASs presented with lesions in the deep brain, including the right thalamus, cingulate gyrus, deep parietal lobe and insular lobe. PET examinations were performed in 14 patients, 12 of which showed focal hypometabolism, one showed hemispheric hypometabolism, and one showed bilateral multiple hypometabolism.

### Differences in clinical characteristics between LGS and non-LGS patients

To further explore the electroclinical difference in ASs between LGS patients and non-LGS patients, we divided the subjects into two groups (9 patients in the LGS group and 7 patients in the non-LGS group). Nine patients were diagnosed with LGS due to multiple seizure patterns (tonic or spasm-dominated) and psychomotor retardation, as well as characteristic EEGs (Fig. 2). All patients diagnosed with LGS demonstrated atypical absence seizures, while 3/7 non-LGS patients exhibited atypical absence seizures, and the other four patients exhibited typical absence seizures (*P* = 0.019, Fisher’s exact test). There was no difference between LGS and non-LGS patients in lesion classification, EEG background, age at seizure onset, age at surgery, seizure onset to surgery interval, intelligence, frequency, or duration of ASs (Table 3).

**Table 3:** The characteristic of LGS and non-LGS in the study

	LGS(9 cases)	non-LGS(7 cases)
AS vs. AAS	0 vs. 9	4 vs. 3*
(Encephalomalacia or encephalatrophy) vs. FCD	6 vs. 3	2 vs. 5
Scalp EEG background (normal vs. abnormal)	1 vs. 8	4 vs. 3

*LGS *Lennox-Gastaut syndrome, *AS *Absence seizures, *AAS *Atypical absence seizures, *FCD *Focal cortical dysplasia

*P = 0.019, Fisher’s exact test

### Surgery and prognosis

All 16 patients underwent surgical resections or dissections (1 hemisphere dissection, 2 hemispherectomies, 4 multilobar resections, and 9 focal resections) and were followed up for 1–9 years (average 4.8 years). All patients had seizure-free outcomes (ILAE I), and their IQ improved by 10.71 ± 3.90 one year after surgery. Pathologically, there were 8 cases of FCD and 8 cases of nonspecific gliosis. The mean number of distinct anti-seizure medications (ASMs) was 3.06, and one patient withdrew from ASM. According to the classification method used in a previous study, postoperative EEGs showed significant improvement in 15 patients compared to preoperative EEGs, and epileptic discharges disappeared in seven patients [15].

## Discussion

Generalized discharges may be present in patients with focal epilepsy on scalp EEG, and generalized or contralateral predominant discharges may occur in either lesion-related LGS or non-LGS patients [10, 16, 17]. Previous studies, including our own, have established that LGS patients exhibiting generalized epileptic discharges on EEG can achieve seizure-free outcomes following precise identification and surgical excision of the epileptogenic focus [9, 10]. These findings suggest that generalized EEG abnormalities may not contradict epilepsy surgery for patients with a focal lesion identified on MRI [9, 10, 16, 18]. Intriguingly, previous studies have demonstrated that seizures in patients with LGS commonly manifest as atypical ASs in addition to generalized tonic seizures. Increasing evidence also suggests that epilepsy related to cortical lesions may manifest as atypical absence seizures, but their specific electroclinical features have not been well documented [5]. In our study, all patients presented with ASs and epileptic lesions, and most also exhibited focal manifestations and preoperative epileptic discharges. All patients had seizure-free outcomes following lesion resection, with marked improvements in psychological assessment and EEG. These results indicated that ASs may rarely be of focal origin and may arise from focal lesions. Moreover, this pattern is not exclusive to LGS patients and can also occur in non-LGS patients.

The ILAE has classified LGS as “generalized combined with focal epilepsy”; however, research indicates that certain generalized seizures in LGS may actually be secondary generalized seizures resulting from focal lesions [5, 19, 20]. Conversely, ASs are classified as typical generalized seizures [1, 21–23]. Furthermore, Bai et al. observed focal changes in blood oxygenation level-dependent functional magnetic resonance imaging (BOLD-fMRI) in focal cortical regions 14 s before seizure onset in childhood absence epilepsy (CAE), which persisted until 10 s after seizure in a specific constant brain region of a single individual [24]. Moreover, studies have suggested that although scalp EEG from patients with childhood absence epilepsy showed bilateral symmetric and synchronous spike-wave discharges (SWDs), the preictal beta and theta band power of EEG presented focal changes, and ictal high-frequency oscillations (HFOs) also predominantly focused on the frontal region [1, 25–27]. Additionally, many fMRI, magnetoencephalography (MEG), and diffusion tensor imaging (DTI) studies have shown focal or local network activity changes preceding ASs, which has also been confirmed in animal models [21, 28–30]. Our results are consistent with the above findings; all patients had absence seizures and epileptogenic lesions, and most also showed focal seizures and focal changes on preoperative EEG. In particular, SEEG showed bilateral synchronous and symmetrical bursts of GSWDs immediately following the focal changes in two patients with simple ASs. And all patients had favorable outcomes after lesion resection. The findings indicate a correlation between lesions and ASs, implying the possibility of a focal onset for ASs. Notably, there are still significant differences between the focal changes of absence seizures and typical focal seizures, such as the speed and range of epileptic discharge propagation and the changes in the width and depth of the network. Thus, it is a practical roadmap to classify ASs as generalized seizures, consistent with the ILAE definition of generalized seizures, which originate from specific points in the brain and rapidly spread to the bilateral brain networks, including cortical and subcortical structures, but not necessarily the entire cerebral cortex [1, 6].

Although the roles of the thalamus and cortex in absence seizures are controversial, the onset of seizures in the cortex is still a focus of significant interest for clinical researchers [1, 21]. Early animal studies showed that the primary sensorimotor cortices may be the site of onset for ASs, and layer 5/6 excitatory pyramidal neurons may be the associated firing neurons [31]. Further clinical studies have shown that electrophysiological focal changes are dominant in the frontal cortex before ASs, especially GSWDs [1, 25]. Moreover, EEG, BOLD-fMRI, and MEG showed that focal changes in the posterior cortex were prominent before ASs, especially in the parietal lobe (the precuneus, posterior cingulate gyrus, lateral parietal lobe, and posterior insular lobe and operculum) [5, 11]. fMRI studies have confirmed that the core network of ASs includes not only the thalamus and striatum but also the medial parietal lobe and lateral parietal lobe, which are important components of the default mode network (DMN) involved in ASs [11]. In this study, the epileptic lesions of nine patients were found to be located in the posterior cortex, while the other three patients had a hemispheric lesion involving the unilateral parietal lobe, which supported the importance of the posterior cortex in the network of ASs [32]. In addition, patients with multiple or extensive lesions also have ASs, suggesting that ASs may have more than one key point in the brain network, which is consistent with Fisher’s speculation that although absence seizures may originate from focal lesions, they are distinct from focal seizures in terms of the range of the affected cortex and deep brain area. Moreover, ASs are involved in many distant cerebral regions, which would be more consistent with the cortical initiation network (CIN) [1, 6].

In our study, the epileptic lesions of four patients with typical ASs were located in deep brain regions, such as the thalamus and insular lobe, indicating that the thalamus plays an important role in the network of ASs, and lesions closer to the thalamus are more likely to cause typical ASs [1, 11]. The thalamus and its reticular neurons are recognized as key links in ASs and the formation of GSWDs [13, 21, 33]. The focal neuronal firing of the thalamocortical circuit directly causes both thalamus and cortical excitations. Thalamic reticular neurons inhibit excitatory transmissions from the cortex and thalamus, resulting in spike waves and slow waves on EEG, respectively, and these effects correspond closely to clinical mild motor symptoms and disruption of consciousness [5, 13, 33]. Therefore, in this study, patients with epileptic lesions in the deep brain regions were more likely to experience typical ASs due to the rapid excitability of thalamocortical circuits.

The present study showed that ASs manifested as a sudden cessation of regular activity and transient disruption of consciousness, which were accompanied by GSWDs on EEG (typical absence seizures), or mild disturbance of consciousness, which was accompanied by tonic seizures, tonic‒clonic seizures, myoclonic seizures and spasms (atypical absence seizures). Intriguingly, patients with ASs complicated with other focal seizures and apparent lesions on MRI had favorable outcomes after lesion resection. Most patients exhibited focal manifestations, focal interictal epileptic discharges, and the ictal focal onset of seizure patterns other than ASs before surgery. SEEG confirmed that focal changes occurred before ASs, so the absence seizures in these patients may still have been of focal origin and soon developed into bilateral generalized seizures, and scalp EEG also showed bilateral synchronous spike and slow-wave complexes. However, the ASs in this study could also be distinguished from focal seizures because they exhibited the semiology and electrophysiological characteristics of absence seizures or atypical absence seizures but did not have focal semiology or scalp EEG origins before epileptic seizures. These results suggested that ASs in patients with lesion-related epilepsy have focal onset with bilateral synchrony, although they seem to be generalized seizures. Furthermore, ASs are usually responsive to ASMs [14]; however, in the study, patients with ASs and lesions did not respond adequately to ASM treatment. Nevertheless, all patients with ASs had seizure-free outcomes after lesion resection, suggesting that ASs should not be a contraindication for surgical resection. Therefore, patients with refractory epilepsy who have brain lesions on MRI, focal symptoms and EEG features in non-absence seizures or SEEG-confirmed focal origins that correspond to the locations of MRI and PET lesions are candidates for surgical intervention. However, this treatment should not be abused, and because of the limitations of current technology and methods, it may be challenging to identify focal changes before ASs and to perform surgery when the onset network is too wide or deep [16].

The limitations of this study include the small sample size, not being a case–control study and the absence of SEEG recordings in most cases. Thus, in many cases, we only described the attributes of the data without performing a statistical analysis. These limitations could potentially influence the generalizability of the findings. Hence, there is a need to include large sample sizes, more SEEG recordings or case‒control studies to validate these results and further explore the mechanisms underlying the relationship between brain lesions and ASs.

## Conclusions

In conclusion, this study indicates that lesion-related epilepsy may rarely be accompanied by absence seizures, and these seizures may have a focal onset and good surgical outcomes after lesion resection.

## Data Availability

All the data and materials are included in the paper.

## Abbreviations

ASs: Absence seizures
EEG: Electroencephalography
ECoG: Electrocorticography
GSWD: Generalized spike-wave discharge
SEEG: Stereoelectroencephalogram
LGS: Lennox-Gastaut syndrome
ILAE: International League Against Epilepsy
FCD: Focal cortical dysplasia
DMN: Default mode network
ASM: Anti-seizure medication
QOLIE: Quality of life in epilepsy
